# Correction: Mechanisms Underlying the Antiarrhythmic Effect of ARumenamide-787 in Experimental Models of the J Wave Syndromes and Hypothermia

**DOI:** 10.1371/journal.pone.0296583

**Published:** 2024-01-02

**Authors:** The PLOS ONE Staff

## Notice of Republication

This article was republished on December 20, 2023, to correct errors in the author byline that were introduced during the typesetting process. The publisher apologizes for the errors. Please download this article again to view the correct version. The originally published, uncorrected article and the republished, corrected articles are provided here for reference.

## Supporting information

S1 File Originally published, uncorrected article. (PDF)

S2 File Republished, corrected article. (PDF)
