# Supplementary material for: Correction: Mechanisms Underlying the Antiarrhythmic Effect of ARumenamide-787 in Experimental Models of the J Wave Syndromes and Hypothermia
Source: PLoS One. 2024 Jan 2;19(1):e0296583. doi: 10.1371/journal.pone.0296583 (PMC10760647; doi:10.1371/journal.pone.0296583)
Supplement: S2 File — (PDF) [file pone.0296583.s002.pdf]

PLOS ONE

RESEARCH ARTICLE

Mechanisms underlying the antiarrhythmic effect of ARumenamide-787 in experimental models of the J wave syndromes and hypothermia

José M. Di Diego[1☯], Hector Barajas-Martinez[1,2☯], Robert Cox[1], Victoria M. Robinson[1], Joseph Jung[1], Mohamed Fouda[3], Bence Patocskai[1], Mena Abdelsayed[3], Peter C. Ruben[3], Charles Antzelevitch[1,2,4]*

1 Lankenau Institute for Medical Research, Wynnewood, PA, United States of America, **2** Sidney Kimmel Medical College, Thomas Jefferson University, Philadelphia, PA, United States of America, **3** Simon Fraser University, Burnaby, BC, Canada, **4** Lankenau Heart Institute, Wynnewood, PA, United States of America

☯ These authors contributed equally to this work.
* cantzelevitch@gmail.com

OPEN ACCESS

Citation: Di Diego JM, Barajas-Martinez H, Cox R, Robinson VM, Jung J, Fouda M, et al. (2023) Mechanisms underlying the antiarrhythmic effect of ARumenamide-787 in experimental models of the J wave syndromes and hypothermia. PLoS ONE 18(5): e0281977. https://doi.org/10.1371/journal.pone.0281977

Editor: Elena G. Tolkacheva, University of Minnesota, UNITED STATES

Received: July 21, 2022

Accepted: February 5, 2023

Published: May 9, 2023

Copyright: © 2023 Di Diego et al. This is an open access article distributed under the terms of the Creative Commons Attribution License, which permits unrestricted use, distribution, and reproduction in any medium, provided the original author and source are credited.

Data Availability Statement: The data have been uploaded to the data repository at Simon Fraser University and can be accessed at the following url: https://summit.sfu.ca/item/35536.

Funding: Supported by National Institutes of Health grants HL47678, HL138103, HL152201 to CA, W. W. Smith Trust to CA and the Wistar and Martha Morris Fund. The funders had no role in study design, data collection and analysis, decision to publish, or preparation of the manuscript.

Abstract

Background

Brugada (BrS) and early repolarization syndromes (ERS), the so-called J wave syndromes (JWS), are associated with life-threatening ventricular arrhythmias. Pharmacologic approaches to therapy are currently limited. In this study, we examine the effects of ARumenamide-787 (AR-787) to suppress the electrocardiographic and arrhythmic manifestations of JWS and hypothermia.

Methods

We studied the effects of AR-787 on I_{Na} and I_{Kr} in HEK-293 cells stably expressing the α- and β1-subunits of the cardiac ($Na_V1.5$) sodium channel and hERG channel, respectively. In addition, we studied its effect on I_{to}, I_{Na} and I_{Ca} in dissociated canine ventricular myocytes along with action potentials and ECG from coronary-perfused right (RV) and left (LV) ventricular wedge preparations. The I_{to} agonist, NS5806 (5–10 μM), I_{Ca} blocker, verapamil (2.5 μM), and I_{Na} blocker, ajmaline (2.5 μM), were used to mimic the genetic defects associated with JWS and to induce the electrocardiographic and arrhythmic manifestations of JWS (prominent J waves/ST segment elevation, phase 2 reentry and polymorphic VT/VF) in canine ventricular wedge preparations.

Results

AR-787 (1, 10 and 50 μM) exerted pleiotropic effects on cardiac ion channels. The predominant effect was inhibition of the transient outward current (I_{to}) and enhancement of the sodium channel current (I_{Na}), with lesser effects to inhibit I_{Kr} and augment calcium channel current (I_{Ca}). AR-787 diminished the electrocardiographic J wave and prevented and/or

Competing interests: I have read the journal's policy and the authors of this manuscript have the following competing interests: Dr. Antzelevitch has served as a consultant for Trevena Pharmaceutical Inc. and has received research funding from Trevena Pharmaceutical, In Carda Pharmaceutical and Kymera Pharmaceutical. The other co-authors have nothing to declare. This does not alter our adherence to PLOS ONE policies on sharing data and materials.

suppressed all arrhythmic activity in canine RV and LV experimental models of BrS, ERS and hypothermia.

Conclusions

Our findings point to AR-787 as promising candidate for the pharmacologic treatment of JWS and hypothermia.

Introduction

The J wave syndromes (JWS) are characterized by distinctive J waves and/or ST segment elevation in specific ECG-leads and are associated with risk for development of polymorphic ventricular tachycardia and fibrillation (pVT and VF) leading to sudden cardiac death (SCD). The two principal forms of JWS are Brugada syndrome (BrS) and early repolarization syndrome (ERS) [1].

First line treatment for high-risk patients suffering from JWS is an implantable cardioverter defibrillator (ICD), an approach that is problematic in young infants for whom a pharmacologic alternative would be preferable. Pharmacological therapy is also desirable for individuals experiencing frequent appropriate ICD shocks [2]. Thus, there is a need for safe and effective pharmacologic treatments capable of preventing life-threatening arrhythmic events associated with JWS. Current pharmacologic approaches to treatment of JWS include the use of quinidine, first suggested by our group in 1999 on the basis of studies conducted in experimental models similar to those employed in the present study [3]. Quinidine's utility is limited by its predisposition to development of an acquired long QT syndrome as well as the fact that it is often not well tolerated at the high doses required due to gastrointestinal complications.

The *KCND3*-encoded $K_V4.3$ transient outward potassium current (I_{to}) plays a pivotal role in the pathophysiology of both syndromes [3,4], owing to its ability to accentuate the action potential (AP) notch and to suppress the epicardial AP dome Accentuation of the action potential notch in the epicardium but not endocardium, secondary to an outward shift in the balance of current in the early phases of the action potential, gives rise to a transmural current that generates an electrocardiographic J wave, which when prominent appears as an ST segment elevation. A further outward shift in the balance of current due to augmentation of I_{to} or reduction of I_{Na} or I_{Ca} leads to loss of the action potential dome at some epicardial sites but not others giving rise to an epicardial as well as transmural dispersion of repolarization, which generates the substrate for the development of phase 2 reentry, giving rise to closely coupled premature beats capable of precipitating pVT/VF).

An ion-channel specific as well as cardio-selective I_{to} blocker and I_{Na} enhancer has long been sought for the management of JWS [2]. The present study tests the hypothesis that a novel compound ARumenamide-787 (AR-787), via its action to inhibit I_{to} and enhance I_{Na}, can effectively suppress the electrocardiographic and arrhythmic manifestations of JWS.

Materials and methods

We examined the effect of AR-787 on: 1) $K_V4.3$ (I_{to}) and $Ca_V1.2$ (I_{Ca}) in canine ventricular epicardial myocytes; 2) $Na_V1.5$ and $K_V11.1$ (hERG—I_{Kr}) in HEK293 cells stably expressing these channels as well as in HEK cells transiently co-transfected with *SCN5A+SCN1B*; and 3) coronary-perfused canine right (RV) and left (LV) ventricular wedge models of JWS in which the I_{to}-agonist, NS5806, alone or in combination with the I_{Ca}-blocker verapamil or the sodium

channel blocker ajmaline were used to elicit the arrhythmic phenotypes by mimicking the genetic defects underlying BrS and ERS.

Canine hearts

This investigation conforms with the Guide for Care and Use of Laboratory Animals. The Lankenau Institute for Medical Research Institutional Animal Care and Use Committee reviewed and approved this research (IACUC Protocol A20-3041, approved Sept. 2, 2021).

Hearts from 20 purpose-bred adult male dogs were used. The first 10 hearts were from mongrel dogs (20–22 kg) acquired from Covance (Denver, PA). Because mongrel dogs were subsequently unavailable, the second set of 10 hearts was from Beagle dogs (10–16 kg) obtained from Envigo (Denver, PA). All dogs were sedated with ketamine (10 mg/kg, IM) and xylazine 2 (mg/kg, IM). Prior to euthanasia (Euthasol solution: pentobarbital sodium and phenytoin sodium; 0.22 ml/kg, IV), heparin (human pharmaceutical grade, 1,000 U/kg, IV) was administered 3–4 min before isolation of the hearts via a left thoracotomy. Retrograde aortic perfusion of the hearts with ice-cold cardioplegic solution (modified K-H solution; 16 mM KCl) was immediately performed to rinse the coronary vasculature of blood. The hearts were then placed in a sealed container containing ice-cold cardioplegic solution and transported to our institution via private courier in an insulated package surrounded by icepacks.

Ventricular wedge preparations

Transmural wedge preparations were dissected from the free wall of the RV or LV. The preparations were cannulated and initially coronary-perfused with cardioplegic solution. The wedge preparations were then placed in a tissue bath and perfused with a modified Krebs-Henseleit solution (K-H) bubbled with 95% O_2/ 5% CO_2 warmed to 37˚C. The composition of K-H was (in mM): 118 NaCl, 4 KCl, 2 $CaCl_2$, 1.2 $MgSO_4$, 24 $NaHCO_3$, 1.2 NaH_2PO_4, 2 Na Pyruvate and 10 D-Glucose. The perfusate was delivered at a constant pressure (45–50 mmHg). Transmembrane action potentials (APs) were simultaneously recorded from the immediate sub-Epi and M cell regions (~2–3 mm from the Endo surface) using floating glass microelectrodes. A transmural pseudo-ECG (ECG) was recorded using two Ag/AgCl half cells placed at ~1 cm from the Epi (+) and Endo (-) surfaces along the same axis as the AP recordings. Pacing was applied to the Endo surface using bipolar silver electrodes insulated except at the tips at Basic Cycle Lengths [BCLs] of 500–2,000 ms. A detailed description of the arterially perfused ventricular wedge preparation has been previously published [5]. ECG and AP signals were digitized and analyzed using Spike 2 for Windows (Cambridge Electronic Design, Cambridge, UK).

Wedge models of the J wave syndromes and hypothermia

Both BrS and ERS have been associated with loss of function variants in the genes that encode the sodium (*SCN5A*, *SCN1B*) and calcium channels (*CACNA1C. CACNB2b*, *CACNA2D1*) as well as gain of function variants in the genes that encode the I_{to} channel (*KCND3*) [2,3]. Accordingly, we used the I_{to}-agonist, NS5806 (NS, 5 or 10 μM), alone or in conjunction with the I_{Ca}-blocker, verapamil (2.5 μM), to pharmacologically mimic the effects of the ion channel genetic defects underlying ERS (left ventricular wedge) and BrS (right ventricular wedge). These provocative agents were added to the coronary perfusate and, when used alone, the concentration of NS was adjusted until the characteristic ECG phenotypes (accentuation of Epi action potential notch and electrocardiographic J waves) were unmasked. AR-787 was subsequently added to the perfusate to suppress the ECG phenotype and prevent the development of arrhythmic activity. In some cases, AR-787 was added after the appearance of phase 2

reentry (P2R), closely coupled premature beats (CCPBs) and/or pVT/VF to test its ability to suppress arrhythmogenesis.

The effects of AR-787 on the ECG and arrhythmic manifestation of hypothermia were studied by reducing the coronary perfusate temperature from 37 to 32˚C [6].

Dissociation of canine ventricular myocytes

Single myocytes were isolated from coronary-perfused canine right or left ventricular wedge preparations via enzymatic dissociation. Briefly, the wedge preparations were coronary perfused with nominally Ca^{+2}-free Tyrode's solution for a period of 15 min. They were then subjected to enzymatic digestion in nominally Ca^{2+}-free solution supplemented with 0.6 mg/ml collagenase (type II; Worthington) and 0.075 mg/ml protease (type XIV; Sigma) for 25–35 min. The wedge preparation was then perfused for 15 min with a taurine buffer solution containing (in mM): 108 NaCl, 10 HEPES, 11 D-glucose, 4 KCl, 1.2 $MgSO_4$, 1.2 NaH_2PO_4, 50 taurine and 0.025 $CaCl_2$. pH was adjusted to 7.4 using NaOH. After perfusion, thin slices of tissue were cut from the epicardial surface to obtain single epicardial cells as previously described [7].

Cell culture

Human Embryonic Kidney (HEK) cells stably expressing hERG channels (NM_000238) were used to evaluate the effect of AR-787 on I_{Kr}. The HEK cells were cultured in vented flasks using standard methods. The culture media consisted of MEM α-modified, 10% FBS, 1% L-glutamine, 1% pen-strep and 0.4% G418 (Geneticin). Cells were passaged at 50% confluence.

HEK cells transiently transfected with wild-type *SCN5A* (α subunit—$Na_V1.5$) together with *SCN1B* (β1 subunit) using Lipofectamine 2000 were used to assess the effect of AR-787 on the cardiac sodium channel current (I_{Na}). All cells were incubated at 37˚C and 5% CO_2. All cell culture reagents were purchased from Thermo Fisher Scientific (Waltham, MA).

Whole-cell patch clamp measurement of I_{Na}, I_{to}, I_{Kr}, I_{Ca} and Action Potentials (APs)

For electrophysiological study, canine ventricular myocytes were released from culture with trypsin, rinsed with Ca^{2+}-free external solution and maintained on ice until used (up to 4 hr). An aliquot of cells was placed in a chamber on an inverted microscope (Nikon Diaphot) and perfused with external solution at a rate of 1 ml/min at room temperature. The external solution contained in (mM): 140 NaCl, 5 KCl, 2 $CaCl_2$, 1 $MgCl_2$, 10 HEPES and 10 glucose at pH 7.4 (adjusted with NaOH). Membrane currents recorded with an Axopatch 200B amplifier controlled by Clampex software were digitized at a sampling rate of 10 kHz and filtered at 2 kHz. Micropipettes were fabricated from borosilicate glass and generally had resistances of 1.8–2.4 MΩ when filled with internal solution consisting of (mM): 140 KCl, 1 $MgCl_2$, 5 Na_2ATP, 10 HEPES and 10 EGTA (pH 7.3 adjusted with KOH; 300 mOsm). Pipette offset and stray capacitance were compensated with the pipette in the bath solution before seal formation. After achieving cell access, whole-cell capacitance and series resistance were compensated optimally. Series resistance prediction and correction were usually adjusted to 85% or higher leaving an uncompensated series resistance of less than 1 MΩ and voltage errors estimated to be less than 5 mV. Voltage protocols and currents were recorded on a computer for offline analysis using Clampfit and GraphPad software.

I_{Na} was recorded from HEK cells co-transfected with *SCN5A* and *SCN1B*. I_{Na} was measured in the voltage-clamp mode in the whole-cell configuration. Whole-cell patch clamp recordings were performed in extracellular solution containing (mM): 140 NaCl, 4 KCl, 2 CaCl2, 1

$MgCl_2$, and 10 HEPES (pH 7.4). Solutions were titrated with CsOH to pH 7.4. Pipettes were fabricated with a P-1000 puller using borosilicate glass (Sutter Instruments, CA, USA), dipped in dental wax to reduce capacitance, then thermally polished to a resistance of 1.0–1.5 MΩ. Low resistance electrodes were used to minimize series resistance between pipette and intracellular solution resulting in typical access resistances of 3.5 MΩ or less, thereby minimizing voltage measurement error. Series resistance compensation to 85% or greater was used to minimize voltage errors to less than 5 mV. Pipettes were filled with intracellular solution. For minimal cytosolic calcium levels, pipettes contained (mM): 130 CsF, 10 NaCl, 10 HEPES, and 10 EGTA titrated to pH 7.4.

All recordings were made using an EPC-9 patch-clamp amplifier (HEKA Elektronik, Lambrecht, Germany) digitized at 20 kHz using an ITC-16 interface (HEKA Elektronik, Lambrecht, Germany). Data were acquired and low-pass-filtered (5 kHz) using PatchMaster/FitMaster software (HEKA Elektronik, Lambrecht, Germany) running on an Apple iMac (Apple Computer, Cupertino, CA). Leak subtraction was performed online using a P/4 procedure. After a giga ohm seal resistance was achieved, the whole-cell configuration was attained.

Current Density. We report current density as the ratio of current amplitude to the cell membrane capacitance (pA/pF).

Activation (GV). To determine the voltage dependence of activation, we measured the peak current amplitude at test pulse potentials ranging from −100 mV to +30 mV in increments of +10 mV for 19 ms. Prior to the test pulse, channels were allowed to recover from fast inactivation at −100 mV for 197 ms. Calculated values for conductance were normalized to the maximal conductance and fit with the Boltzmann function:

$$G/G_{max} = 1/(1 + \exp[-ze_0[V_m - V_{1/2}]/kT])$$

where G/G_{max} is the normalized conductance amplitude, V_m is the command potential, z is the apparent valence, e_0 is the elementary charge, $V_{1/2}$ is the midpoint voltage, k is the Boltzmann constant, and T is temperature in ˚K.

Steady-State Fast Inactivation (SSFI). The voltage-dependence of SSFI was measured by preconditioning the channels to a hyperpolarizing potential of −170 mV and then eliciting prepulses from −170 to +30 mV in increments of 10 mV for 500 ms. Channel availability was assessed during a test pulse to 0 mV. Normalized current amplitude as a function of voltage was fit using the Boltzmann function:

$$I/I_{max} = 1/(1 + \exp(-ze_0(V_m - V_{1/2})/kT)$$

where I/Imax is the normalized current amplitude, z is apparent valence, e_0 is the elementary charge, Vm is the prepulse potential, V1/2 is the midpoint voltage of SSFI, k is the Boltzmann constant, and T is temperature in ˚K.

Fast Inactivation Kinetics. Time constants for open-state fast inactivation were derived by fitting a single exponential function to the decay of current obtained from the activation protocol.

$$I = I_{ss} + \alpha \exp(-(t - t_0)/\tau)$$

where I is current amplitude, Iss is the plateau amplitude, α is the amplitude at time 0 for time constant τ, and t is time.

Persistent I_{Na} Current. The early and late components of the persistent I_{Na} were measured during a 10 ms and a 200 ms depolarizing pulse to 0 mV from a holding potential of −130 mV.

I_{to} and APs were measured in isolated RV or LV myocytes. APs were measured in the current-clamp mode in the whole-cell configuration. APs were initiated using intracellular

current injection and recorded continuously by means of Clampex software (Axon Instruments/Molecular Devices) at a sampling frequency of 0.5 Hz. I_{to} was measured using the Axopatch 200B-2 amplifier in V-Clamp mode.

I_{to} was recorded in isolated ventricular epicardial cells at 37˚C using whole-cell patch clamp techniques. I_{to} was elicited using a series of 350 ms voltage steps between -50 mV and +40 mV from a holding potential of -90 mV. A 15 ms prepulse to -20 mV was used to discharge the sodium current. Interpulse interval was 50 ms. The amplitude of I_{to} was determined by subtracting peak current from steady-state current at the end of the pulse. The total charge carried by I_{to} was determined by calculating the area under the I_{to} current waveform. The extracellular solution contained (in mM): 140 NaCl, 4 KCl, 1.8 $CaCl_2$, 1.2 $MgCl_2$, 10 HEPES, and 10 glucose, pH 7.4 (adjusted with NaOH). The intracellular solution contained (in mM): 120 KCl, 1.5 $MgCl_2$, 10 HEPES, 0.5 EGTA, 0.01 $CaCl_2$, 5 MgATP, pH 7.2 (adjusted with NaOH). Following baseline measurements, recordings was repeated during exposure to AR-787.

I_{Ca} was measured in canine ventricular myocytes at 37˚C. I_{Ca} was elicited by application of 300 ms steps from -50 to +40 mV. Extended protocols were avoided to prevent current rundown. Internal solution contained (in mM): 120 CsCl, 1 $MgCl_2$, 10 EGTA, 5 Mg-ATP, 10 HEPES, 5 $CaCl_2$. pH was adjusted to 7.2 with CsOH. External solution contained (in mM): 140 NaCl, 10 HEPES, 10 D-glucose, 4 KCl, 1 $MgCl_2$, 2 $CaCl_2$, 2 4-AP and 0.1 $BaCl_2$.

I_{Kr} was measured using a two-step protocol. Voltage steps of 4 sec duration were applied at 20 sec intervals from a holding potential of -80 mV to -60 to +40 mV in 10 mV steps, followed by a 4 sec step to -50 mV to record the tail currents. Following control recordings, a step protocol was applied, and perfusion changed electronically (ALA Scientific) to an external solution containing 10 μM AR-787. The step protocol consisted of a voltage step from a holding potential of -80 mV to a test potential of +10 mV for 4 sec then to -50 mV for 4 second. It was repeated at 20 sec intervals. The step protocol was maintained until the current response achieved a stable level (5–7 min). The I-V protocol was then repeated.

For analysis, the following were determined: 1) currents averaged over the last 100 ms of the first voltage clamp step (-60 to +40 mV) and 2) peak tail current during the second voltage clamp step (at -50 mV). These were divided by cell capacitance to normalize for cell size (pA/pF). The time course of tail current inactivation was fit with a single exponential function using Clampfit. The voltage dependence of activation was determined from tail currents by dividing current value at each voltage by the maximum value for each I-V protocol. Values of current were averaged among cells and expressed as a mean ± SEM.

Drugs

ARumenamide-787 (AR-787 [MolPort-005-972-787/ZINC000012323863/Vitas-MSTK638098]) was purchased from MolPort SIA (Riga, Latvia) in powder form. It was dissolved in 100% DMSO to create a stock solution of 50 mM and diluted with external solution to the desired final concentration. DMSO content was always 0.1% or less. Verapamil and NS5806 were purchased from Sigma-Aldrich (St Louis, MO). Stock solutions of all drugs were prepared in 100% DMSO. Drug-response relationships were determined by introducing increasing concentrations of the pharmacological agent to different groups of cells. Thus, each cell was exposed to a single concentration of AR-787.

Statistical analysis

Statistical analysis was performed using one-way repeated measures analysis of variance (ANOVA) or a two-factor completely randomized design ANOVA followed by a post hoc Tukey test. Our statistical model was a full factorial in which all the factors were allowed to

interact together. Statistical differences in voltage clamp analysis were evaluated using Student's paired single-sided *t*-test. Data are shown as mean ± SEM. Statistical significance was considered at $p<0.05$.

Intellectual property

AR-787, and other compounds of the same class, are under IP protection (Publication Number WO/2020/161606; International Application No. PCT/IB2020/050853) and Lankenau Institute for Medical Research + Simon Fraser University provisional patent application submitted October 1, 2021.

Results

Fig 1 illustrates the molecular structure of Arumenamide-787 (AR-787) and the effect of AR-787 on I_{to}, I_{Ca} and action potential morphology recorded from enzymatically dissociated canine RV epicardial myocytes [8]. AR-787 (10 μM) significantly suppressed the AP notch and produced a concentration-dependent inhibition of I_{to}. At +20 mV, I_{to} density was 21.79 +/-3.06 pA/pF in control. At concentrations of 1, 10 and 50 μM, AR-787 reduced I_{to} density by 34% (to 14.34+/-2.42 pA/pF), 68% (to 7.00+/-2.80 pA/pF) and 85% (to 3.23+/-2.20), respectively (Fig 1B and 1C). Action potential duration at 90% repolarization (APD_{90}) did not change significantly (403.0±10.3 vs. 390.6±8.3 ms). The IC_{50} for inhibition of I_{to} is estimated to be approximately 8 μM. AR-787 (10 μM) increased I_{Ca} peak density from -5.13+/-0.77 pA/pF to -6.95+/-1.88 pA/pF at 0 mV (Fig 1D and 1E); the shift in the I-V curve, however, was not statistically significant.

Quinidine, the drug currently recommended for use in JWS, in addition to blocking I_{to}, also inhibits I_{Kr}, which can potentially result in an acquired long QT phenotype. We were therefore interested in assessing whether AR-787 also possess this undesirable action**.** Fig 2 shows the effect of AR-787 on I_{Kr} measured in HEK cells stably expressing hERG (n = 8). The amplitude of the developing I_{Kr} was enhanced between -60 to -20 mV and reduced above 0 mV. These effects resulted in a very minor net increase of I_{Kr} at voltages associated with action potential repolarization consistent with the observation that APD and QT interval were largely unaffected.

The effect of AR-787 on I_{Na} was measured in HEK cells co-transfected with *SCN5A* +*SCN1B* (**Fig 3**). AR-787 (10 μM) significantly enhanced sodium current by approximately 4- and 7-fold at -50 and -40 mV, respectively (Fig 3B and Table 1). However, the drug had no effect on the voltage-dependence of activation and steady-state fast inactivation (Fig 3 and Table 1). The early and late components of the persistent sodium current (%) underlying phases 1–2 of the cardiac AP were unaltered by AR-787 (10 μM). The kinetics of fast inactivation were significantly accelerated by approximately 1.4-folds by AR-787 (10 μM) at -20 mV and 0 mV (Table 1).

In another experimental series, we used canine ventricular RV and LV wedge preparations to generate experimental models of JWS by pharmacologically mimicking the effects of the associated genetic defects leading to an increase of I_{to} and/or to reduced levels of I_{Na} or I_{Ca}. Table 2 shows the effects of the I_{to} agonist NS5806 (NS, 5–10 μM) and of AR-787 (AR, 10 μM) on action potential and ECG parameters (BCL = 800 ms; LV wedges (n = 3). The introduction of 5–10 μM NS5806 to the coronary perfusate increased the magnitude of phase 1, which accentuated the Epi AP notch and electrocardiographic J wave, thus leading to the development of the JWS phenotype. AR-787 (10 μM) readily reversed these effects in all cases.

Fig 4 shows the effect of 1, 10 and 50 μM AR-787 to suppress the electrocardiographic (ECG) manifestations of JWS in models of ERS and BrS generated by exposing LV (**Panels A**

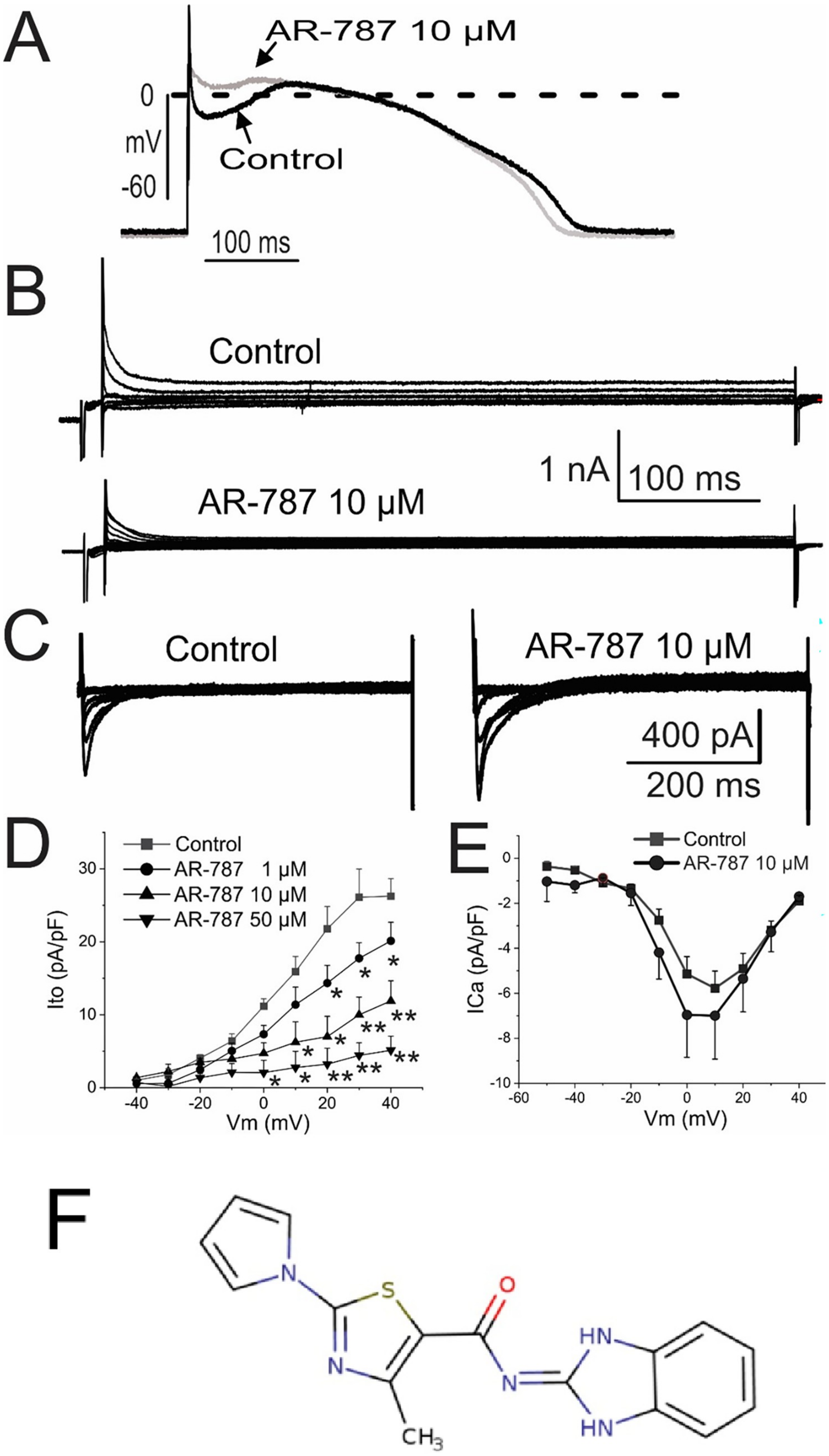
A
AR-787 10 μM
0
mV
-60
Control
100 ms
B
Control
1 nA
100 ms
AR-787 10 μM
C
Control
AR-787 10 μM
400 pA
200 ms
D
Control
AR-787 1 μM
AR-787 10 μM
AR-787 50 μM
Ito (pA/pF)
Vm (mV)
E
Control
AR-787 10 μM
ICa (pA/pF)
Vm (mV)
F
S
N
N
CH3
O
N
HN
HN

Fig 1. Effect of AR-787 on the transient outward current (I_{to}), inward calcium current (I_{Ca}) and action potential recorded from canine right ventricular epicardial myocytes. (n = 6) A: Representative action potential traces recorded in the absence (Control) and presence of AR-787 (10 μM) at a frequency of 0.5 Hz. **B** and **C**: Representative traces of I_{to} and I_{Ca} recorded in Control and following exposure to AR-787 (10 μM). **D** and **E:** I-V relationships for peak I_{to} and I_{Ca} recorded under control conditions and following exposure to AR-787. **F:** Molecular structure of Arumenamide-787 (AR-787). All data were obtained at 37˚C. Data are reported as mean ± SEM; *p<0.05, ** p<0.001 vs. Control (One Way Repeated Measures ANOVA).

https://doi.org/10.1371/journal.pone.0281977.g001

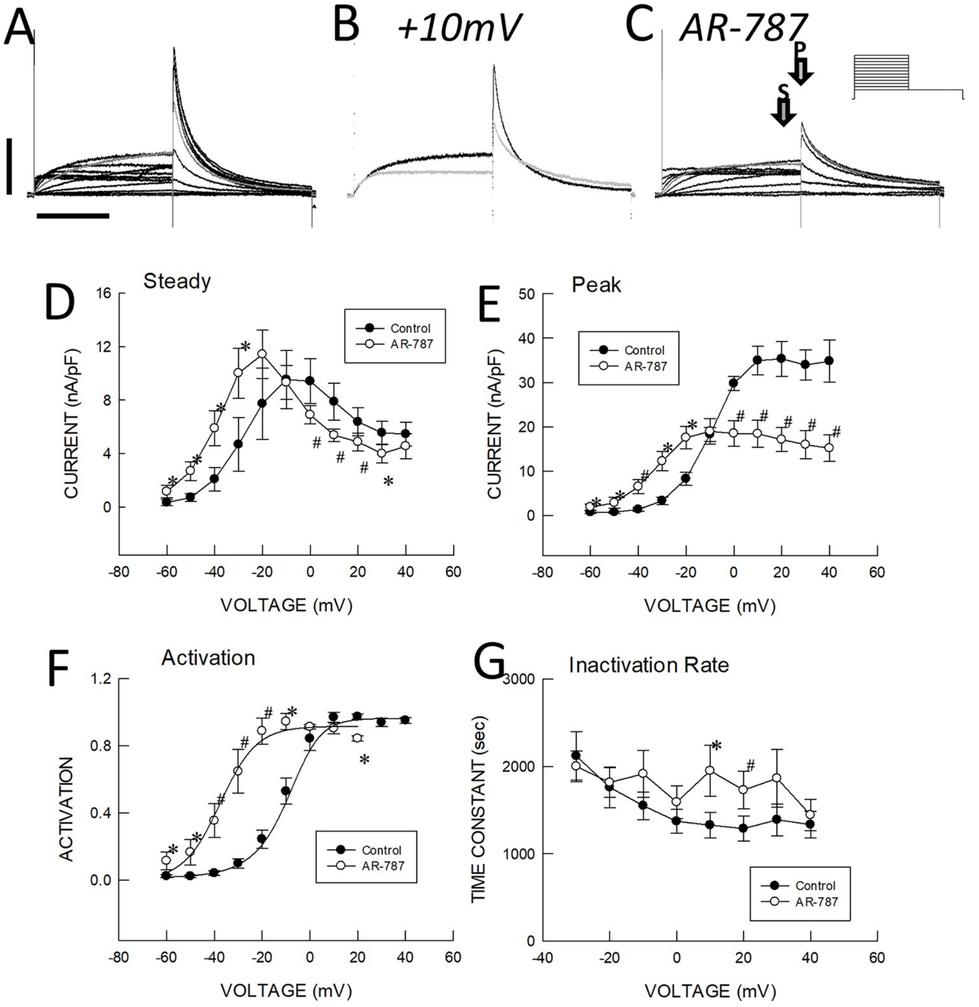

Fig 2. Effects of 10mM AR-787 on hERG currents. A: Control currents recorded prior to addition of AR-787. **B:** Currents recorded at a test potential of +10mV before (control, black) and 7 min after the addition of AR-787 to the perfusate (grey). **C:** Currents recorded after AR-787 addition (protocol started 7 min after the initiation of AR-787 addition). **D:** Voltage dependence of steady currents recorded at last 50 msec of the first voltage step (arrow labeled S in panel C). **E:** Voltage dependence of peak tail currents recorded at -50mV (arrow labeled P in panel C). **F:** Voltage dependence of activation determined from tail currents normalized to the peak value for each test potential. **G:** Inactivation time constant determined by curve fitting a single exponential to the tail current. Grey traces in A and C are recorded at a test potential of +10mV. Vertical line below panel A represents 0.7nA while horizontal line represents 2 sec. Data points and vertical bars in panels D-G represent mean ±1 SEM (n = 8). Symbols above or below data points in panels D-G represent statistically significant differences between control and AR-787 determined by a paired data, single-sided Student's t-Test at p<0.05 (*) or p<0.001 (#).

https://doi.org/10.1371/journal.pone.0281977.g002

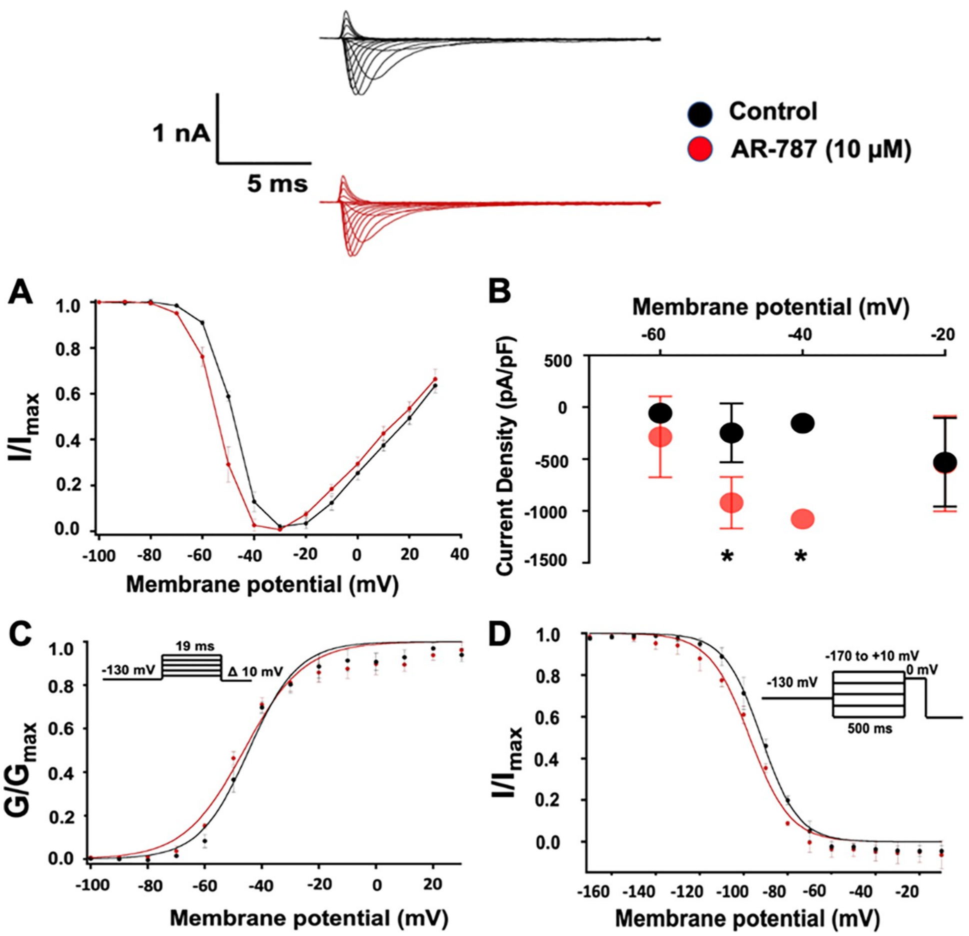

Fig 3. The effect of AR-787 on sodium channel activity. The upper panel shows representative traces of the effect of AR-787 (10 μM) on I_{Na} (AR-787 -red and Control—*black*). **A:** AR-787 (10 μM) induced a significant increase in the conductance of sodium as clearly seen in the significant increase in I_{Na} density **(B)** at -50 mV and -40 mV. **C:** AR-787 (10 μM) has no significant effect on the voltage-dependence of conductance. **D:** AR-787 (10 μM) has no significant effect on the voltage-dependence of steady-state fast inactivation.

https://doi.org/10.1371/journal.pone.0281977.g003

and **B**) and RV (**Panel C**) wedge preparations to 10 μM of NS5806. From top to bottom, all columns (Col) in each panel show simultaneously recorded transmembrane action potentials from the M and Epi regions together with a pseudo-ECG. Fig 4 shows recordings obtained under control conditions, 20–40 min of exposure to 10 μM NS5806 and following further addition of 1–50 μM AR-787 to the coronary perfusate.

The addition of NS5806 to the coronary perfusate dramatically augmented the Epi AP notch resulting in the accentuation of the J wave in the ECG, but without the development of arrhythmias (**Col 2** of Fig 4A, 4B and 4C). Fig 4C shows loss of the RV Epi action potential dome after 40 min of exposure to the I_{to} agonist NS5806 (10 μM). AR-787 caused a concentration-dependent diminution in the manifestation of the AP notch and associated ECG J wave. Although not shown in the figure, it is noteworthy that several episodes of Phase 2 reentry, manifesting as closely coupled extrasystoles, developed following 40 min of exposure to NS5806. AR-787 (50 μM) was effective in fully restoring the AP dome and in totally suppressing all arrhythmic activity.

Fig 5 summarizes the effect of AR-787 (10 μM) following NS5806 (5–10 μM) on AP and ECG parameters (also reported in Table 2) recorded from LV wedge preparations (n = 3).

Table 1.

	Control	AR-787	*n*	*P-value*
	(Mean ± SEM)	*(Mean ± SEM)*		
**GV–$V_{1/2}$ (mV)**	-44.2 ± 2.2	-50.1 ± 2.5	4	0.1244
**GV–z (slope)**	4.1 ± 0.2	3.9 ± 0.2	4	0.4502
**SSFI–$V_{1/2}$ (mV)**	-90.7 ± 3.9	-95.6 ± 4.4	4	0.4339
**SSFI–z (slope)**	-3.5 ± 0.1	-3.6 ± 0.2	4	0.6324
**I_{Nap} (10 ms)**	6.3 ± 0.1	4.8 ± 0.3	4	0.2545
**I_{Nap} (200 ms)**	0.8 ± 0.1	0.8 ± 0.1	4	0.8395
**Current density (pA/pF)**				
**-60 mV**	-57.4 ± 20.3	-285.8 ± 194.8	4	0.2286
**-50 mV**	-246.7 ± 141.8	-921.2 ± 124.6	4	0.0223*
**-40 mV**	-154.2 ± 23.0	-1077.2 ± 33.3	4	<0.0001*
**-20 mv**	-505.5 ± 227.5	-532.3 ± 248.2	4	0.9535
**Tau of inactivation**				
**-20 mV**	1117.9 ± 50.8	788.8 ± 38.1	4	0.0020*
**0 mV**	812.6 ± 32.2	630.8 ± 21.5	4	0.0034*
**10 mV**	661.0 ± 62.8	572.0 ± 20.4	4	0.2265

https://doi.org/10.1371/journal.pone.0281977.t001

Addition of AR-787 (10 μM) to the coronary perfusate readily reversed the effect of NS5806 to augment phase 1 magnitude of the epicardial AP (Epi Ph1 magnitude) as well as the dramatic increase in J wave amplitude. QT interval was unaffected by either drug.

Fig 6 shows the effect of AR-787 to suppress ECG and arrhythmic manifestations in a wedge model of BrS in which the disease phenotype was provoked using NS5806 to mimic the gain of function of I_{to} and ajmaline to mimic the loss of function of I_{Na} caused by genetic defects associated with BrS. The I_{to} agonist greatly amplified the J wave in the ECG causing the typical coved type Brugada sign. The addition of ajmaline led to loss of the action potential dome in the epicardium but not in the M region, thus creating transmural dispersion of repolarization, which led to the development of phase 2 reentry and pVT (Fig 6C). The addition of AR-787 (10 μM, Fig 6D) restored the action potential dome in the epicardial action potentials via its effect to inhibit I_{to} and enhance I_{Na}, thus restoring homogeneity of repolarization

Table 2. The effect of NS5806 (NS, 5–10 μM) and AR-787 (10 μM) on action potential and ECG parameters in models of JWS.

CL 800 msec	Control			NS (5–10 μM)			AR (10 μM)		
M (APD50)	196.4	±	5.9	179.5	±	5.9	179.0	±	11.4
Epi (APD50)	174.7	±	8.1	181.8	±	10.4	173.9	±	7.8
M (APD90)	252.1	±	8.4	228.4	±	7.7	235.0	±	11.1
Epi (APD90)	211.6	±	8.0	215.6	±	5.3	209.0	±	8.1
Ph1 mag (%Ph0)	18.8	±	7.9	40.2	±	6.5*	25.4	±	4.9
QT interval	252.5	±	8.3	261.2	±	4.5	254.3	±	10.0
T wave amplitude	22.9	±	5.8	17.8	±	9.6	19.0	±	1.8
J wave amplitude	10.9	±	4.6	31.3	±	4.6*	8.7	±	5.7

Left: Data (M ± SE) derived from 3 LV wedge preparations. BCL = 800 msec. Right: Illustration of an epicardial AP, showing the basis for measurement of Phase 1 (Ph1) magnitude (measured as a % of the amplitude of Phase 0), J wave amplitude (quantified from its onset to the peak), and QT interval (measured from the onset of the QRS to the end of T wave determined by the tangent method [intersection of the maximum downslope of the T wave to the isoelectric line]).

* p<0.005 vs. Control and AR-787 (One Way RM ANOVA).

https://doi.org/10.1371/journal.pone.0281977.t002

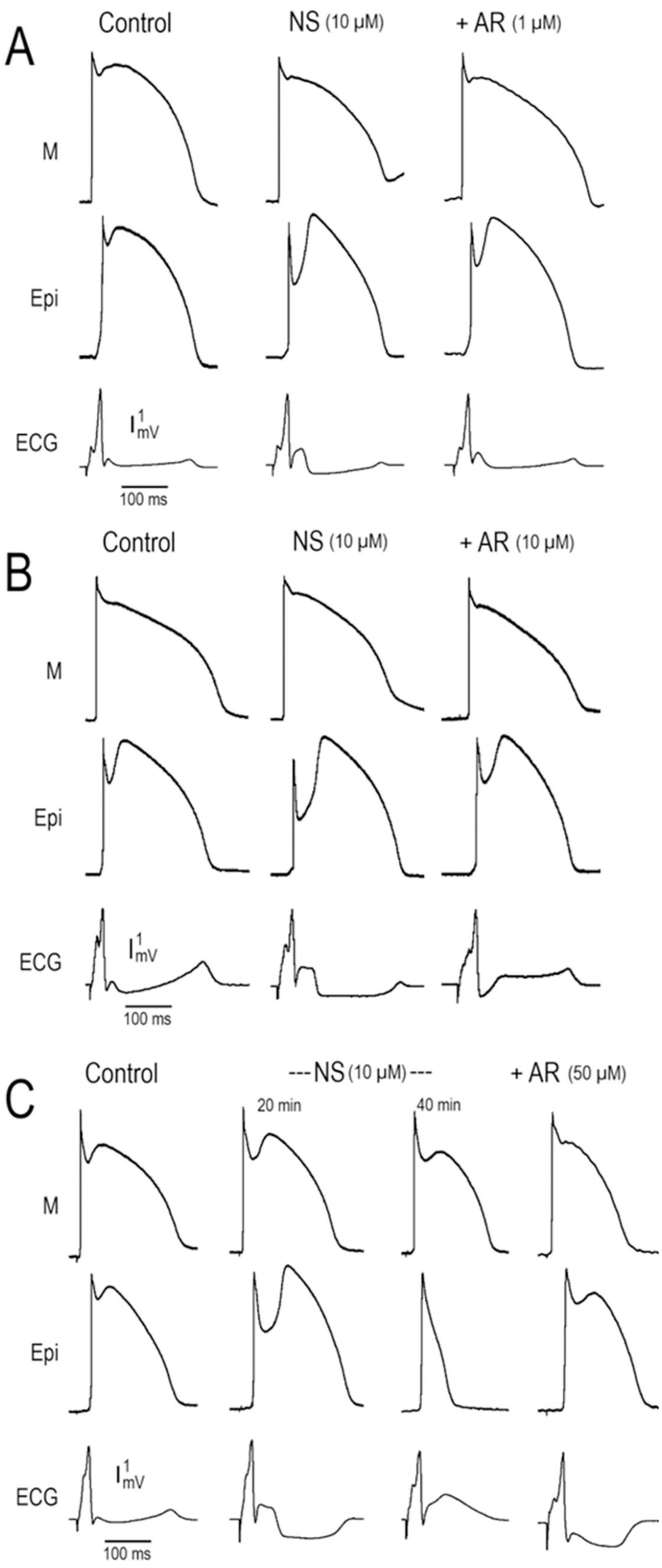
A
Control
NS (10 μM)
+ AR (1 μM)
M
Epi
ECG
1 mV
100 ms
B
Control
NS (10 μM)
+ AR (10 μM)
M
Epi
ECG
1 mV
100 ms
C
Control
---NS (10 μM)---
+ AR (50 μM)
20 min
40 min
M
Epi
ECG
1 mV
100 ms

Fig 4. Effect of 1, 10 and 50 μM of AR-787 to suppress the electrocardiographic manifestations in models of JWS (ERS and BrS). From top to bottom, all columns (Col) in each panel shows simultaneously recorded transmembrane action potentials from the M and Epi regions together with a pseudo-ECG from 3 different wedge preparations (panels A [LV wedge 1], B [LV wedge 2] and C [RV wedge]). BCL = 800 ms. **A:** Recording obtained under Control (Col 1), after 20 min of perfusion with NS5806 (NS, 10 μM) (Col 2) and **25 min** after addition of AR-787 (AR, **1 μM**) to the perfusate (Col 3). **B:** Recordings obtained under Control conditions (Col 1), 20 min after perfusion with NS5806 (NS, 10 μM) (Col 2) and **20 min** after addition of AR-787 (AR, **10 μM**) (Col 3). **C:** Recordings obtained under Control conditions (Col 1), after 20 and 40 min of exposure to NS5806 (NS, 10 μM) (Col 2 and Col 3, respectively) and **15 min** after addition of AR-787 (AR, **50 μM**) to the coronary perfusate (Col 4).

https://doi.org/10.1371/journal.pone.0281977.g004

throughout the preparation, resulting in suppression of all arrhythmic activity. Washout of AR-787 resulted in reappearance of the BrS phenotype with restoration of a prominent J wave in the ECG due to loss of the epicardial AP dome (Fig 6E).

Fig 7 shows the effect of AR-787 to suppress the electrocardiographic and arrhythmic manifestations of JWS in a wedge model of ERS in which the disease phenotype was provoked using NS5806 to mimic the gain of function of I_{to} and verapamil to mimic loss of function of calcium channel current (I_{Ca}), both of which have been shown to be associated with genetic defects causing ERS. Exposure of the in a **LV wedge** to NS5806 and verapamil resulted in the development of a prominent J wave and a closely coupled extrasystole following loss of the epicardial action potential dome (**Panel B**), most likely due to phase 2 reentry, which subsequently triggered pVT (**Panel C**). AR-787 (10 μM) reduced Phase 1 magnitude, restored the AP dome and totally suppressed all arrhythmic activity (**Panel D**). These effects of the drug were observed within minutes of its introduction to the coronary perfusate. AR-787 was effective in suppressing all of the electrocardiographic and arrhythmic manifestations of JWS (**Figs 4–6**).

Fig 8 shows the effect of AR-787 to suppress ECG and arrhythmic manifestations in a wedge model of ERS in which the disease phenotype was provoked using NS5806 to mimic the

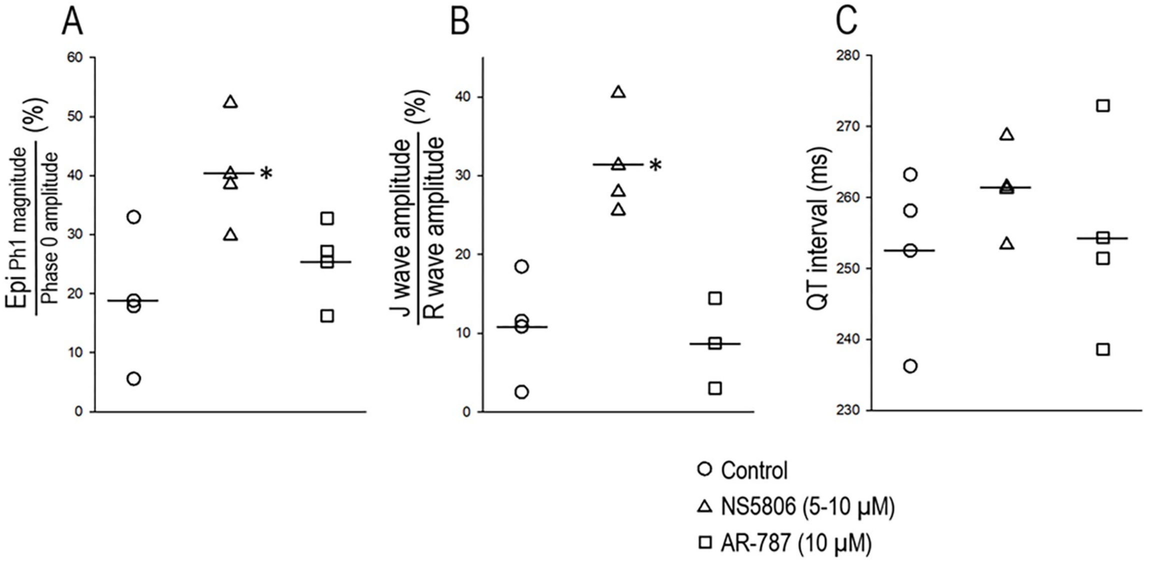

Fig 5. Effect AR-787 (10 μM) following NS5806 (5–10 μM) on AP and ECG parameters recorded from 3 LV wedge preparations. A: Epi Ph1 magnitude as a % of Ph0 amplitude. **B:** J wave amplitude as a % of R wave amplitude. **C:** QT interval (ms). BCL = 800 ms (LV wedges; n = 3). NS5806 (5–10 μM) significantly increased the Epi Ph1 magnitude and the simultaneously recorded electrocardiographic J wave. AR-787 (10 μM) reversed this effect restoring these parameters to control values. The QT interval was unaffected by either intervention. The symbols are data points, and the crossed symbols indicate the mean values (data derived from Table 2); * $p<0.005$ vs. Control and AR-787 (One Way Repeated Measures ANOVA).

https://doi.org/10.1371/journal.pone.0281977.g005

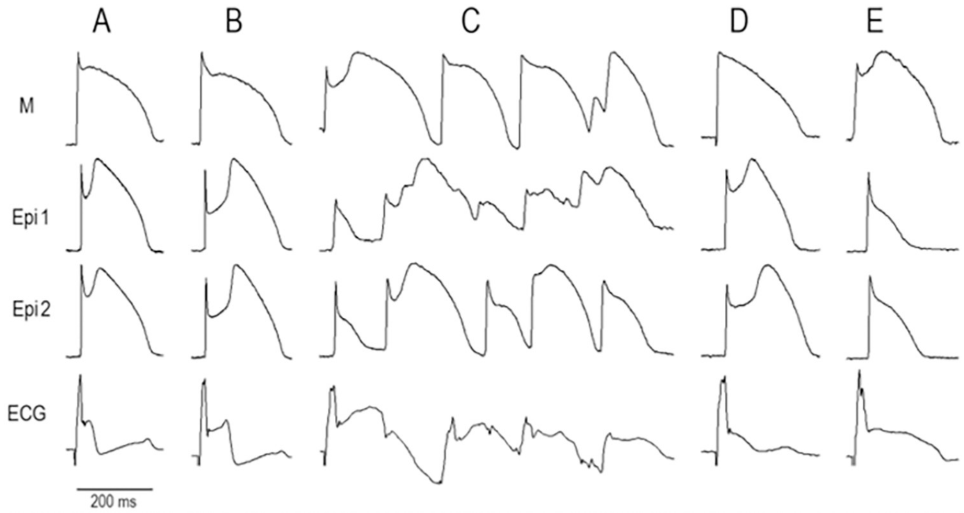

Fig 6. AR-787 suppresses ECG and arrhythmic manifestations in a wedge model of BrS in which the disease phenotype was provoked using ajmaline to mimic the loss of function of sodium channel currents caused by mutations in genes encoding the cardiac sodium channel. From top to bottom, each panel shows action potentials recorded from the M region and 2 epicardial sites (Epi 1 and Epi 2) of a **RV wedge** preparation together with a pseudo-ECG (ECG). BCL: 2000 ms **A:** Control. **B:** Recordings obtained after addition of NS5806 (6 μM; 45 min) to the coronary perfusate. **C:** Further addition of ajmaline (2.5 μM; 30 min) dramatically increased the J wave leading to development of a short run of pVT triggered by a closely coupled premature beat arising from Epi (Phase2-reentry). **D:** Addition of AR-787 (10 μM; 20 min) restored the epicardial AP dome, greatly diminished the J wave in the ECG and suppressed all arrhythmic activity. **E:** Washout of AR-787 (85 min) resulted in reappearance of the BrS phenotype with restoration of a prominent J wave in the ECG due to loss of epicardial AP dome.

https://doi.org/10.1371/journal.pone.0281977.g006

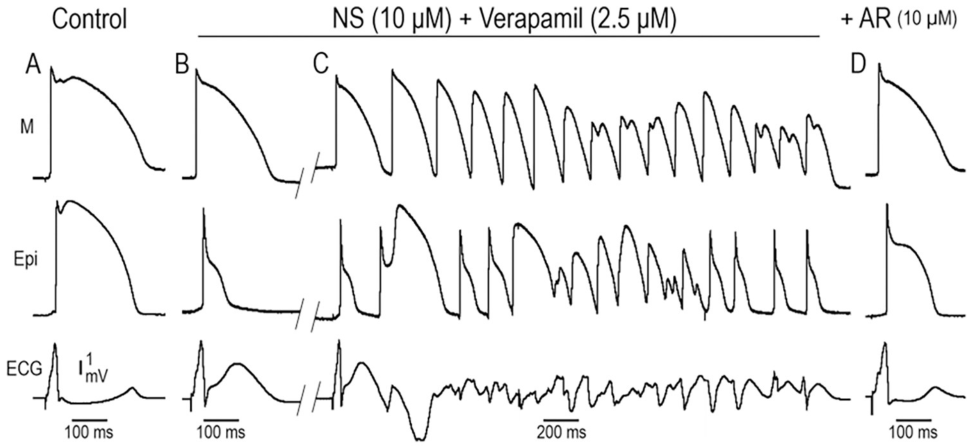

Fig 7. Effect of AR-787 to suppress the electrocardiographic and arrhythmic manifestations in a model of ERS in which the disease phenotype was provoked by exposure to the I_{to} agonist, NS5806, and the calcium channel blocker, verapamil. From top to bottom, each panel shows simultaneously recorded transmembrane action potentials from the M and Epi regions together with a pseudo-ECG from a LV wedge preparation. BCL = 800 ms. **A:** Control recordings. **B:** Recordings obtained after 40 min of perfusion with a combination of NS5806 (NS, 10 μM) and verapamil (2.5 μM) **C:** Recordings obtained 22 min after the recordings shown in panel B. A closely coupled premature extrasystole is seen to trigger a short run of pVT. **D:** Recordings obtained 30 min after the addition of AR-787 (AR10 μM) to the coronary perfusate.

https://doi.org/10.1371/journal.pone.0281977.g007

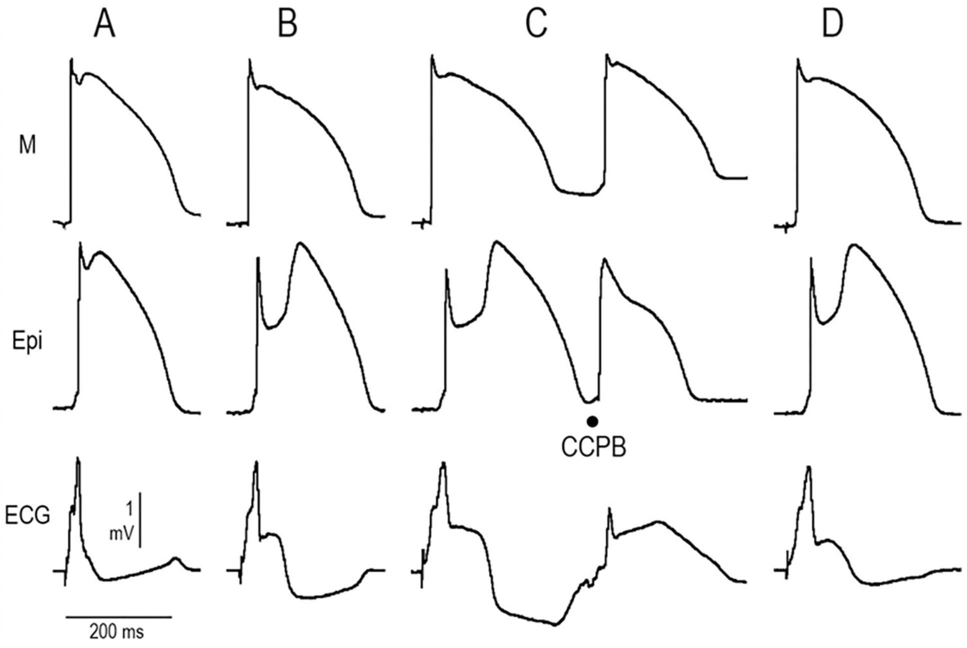

Fig 8. AR-787 suppresses the ECG and arrhythmic manifestations in a wedge model of ERS in which the disease phenotype was provoked using NS5806 and ajmaline. From top to bottom, each panel shows M and Epi APs and a pseudo-ECG (ECG) recorded from a **LV wedge** preparation. BCL: 2000 ms. **A:** Control. **B:** Recordings obtained after addition of NS5806 (3.75 μM; 60 min) to the coronary perfusate. **C:** Further addition of ajmaline (2.5 μM; 40 min) dramatically increased the J wave, leading to development of a closely coupled premature beat (CCPB) arising from Epi, consistent with a phase 2-reentry mechanism. **D:** Further addition of AR-787 (10 μM; 5 min) reduced the manifestation of the epicardial AP notch and J wave in the ECG and suppressed all arrhythmic activity.

https://doi.org/10.1371/journal.pone.0281977.g008

Table 3. Effect of AR-787 to Suppress Phase 2 Reentry (P2R) and polymorphic VT (pVT).

JWS Model	Provocative Agents	P2R	pVT
Brugada Syndrome (RV Wedge)	N5806, Verapamil	2	1
	+AR-787	0	0
	NS5806, Ajmaline	2	2
	+ AR-787	0	0
	NS5806	1	1
	+ AR-787	0	0
Hypothermia (RV wedge)	N5806, Hypothermia	1	1
	+AR-787	0	0
Early Repolarization Syndrome (LV Wedge)	N5806, Verapamil	1	1
	+AR-787	0	0
	NS5806, Ajmaline	1	1
	+ AR-787	0	0
	NS5806	1	1
	+ AR-787	0	0
Hypothermia (LV wedge)	N5806, Hypothermia	2	2
	+AR-787	0	0

Numbers denote the number of preparations in which these results were observed.

https://doi.org/10.1371/journal.pone.0281977.t003

gain of function of I_{to} and ajmaline to mimic the loss of function of sodium channel currents caused by mutations in genes encoding the cardiac sodium channel. The I_{to} agonist greatly amplified the J wave in the ECG. The addition of ajmaline led to the development of a closely coupled extrasystole in epicardium, likely due to a phase 2 reentrant mechanism. The addition of AR-787 (10 μM) reduced the epicardial AP notch and the J wave in the ECG, leading to suppression of all arrhythmic activity (**Fig 8D**).

As we previously reported, therapeutic hypothermia (32˚C) produces ECG and arrhythmic manifestation similar to those of BrS and ERS [6]. In 2 LV and one RV wedge preparations we tested the ameliorative effects of AR-787 in the setting of hypothermia. In all cases AR-787 was effective in suppressing the development of prominent J waves, phase 2 reentry and pVT induced by hypothermia (32˚C).

Table 3 summarizes the effectiveness of AR-787 to suppress phase 2 reentry and pVT in the various models of BrS, ERS and hypothermia tested.

Discussion

The search for an ion-channel specific and cardio-selective I_{to} blocker and/or I_{Na} enhancer capable of mediating therapy for a variety of inherited cardiac arrhythmia syndromes is now in its third decade [2,3]. The present study tests the hypothesis that AR-787, via its action to inhibit I_{to} and enhance I_{Na} can effectively suppress the electrocardiographic and arrhythmic manifestations of JWS. Our findings point to AR-787 as a promising new agent for pharmacologic therapy of JWS due largely to its unique effect to block I_{to} and enhance I_{Na}, and thus prevent repolarization abnormalities underlying arrhythmogenesis in patients with JWS.

Our study demonstrates the effect of AR-787 at concentrations as low as 10 μM to suppress the electrocardiographic and arrhythmic manifestations of both BrS and ERS as well as of hypothermia. Our findings add to the long list of studies providing support for the hypothesis that an inward shift in the balance of currents contributing to the early phases of the ventricular AP exerts ameliorative effects on the electrocardiographic and arrhythmic manifestations of JWS. Augmenting this inward depolarizing reserve reverses the effects of the outward shift in the balance of currents in the early phase of the epicardial action potential caused by the genetic defects underlying in JWS [6,9–16]. Using experimental approaches similar to those utilized in this study, we previously reported the benefits of inhibiting outward currents such as I_{to} with agents such as quinidine and acacetin, augmenting inward calcium channel current (I_{Ca}) using drugs such as isoproterenol, as well as combined inhibition of I_{to} and augmentation of I_{Ca} using phosphodiesterase inhibitors such as cilostazol and milrinone in BrS and ERS [13]. AR-787 produces an inward shift in the balance of current not only by suppressing I_{to}, but also by enhancing I_{Na} and to a lesser extent I_{Ca} during the early phases of the action potential. The effect of AR-787 to enhance I_{Na} is similar to that previously described by us for lithospermate B, which suppresses arrhythmogenesis in experimental models of the J wave syndromes [6,17,18]. These effects of the pharmacological agents showed an onset within minutes as well as reversal of their ameliorative effects upon washout, supporting the conclusion that the observed electrophysiological changes were due to exposure to the drug and not to time-dependent changes.

In the clinic, ERS and BrS share similarities in their response to pharmacological therapy. Electrical storms (and the associated accentuated J waves) can be suppressed with β-adrenergic agonists [19–22]. Chronic oral administration of quinidine [23,24], bepridil [21], denopamine [19,25], and cilostazol [19,21,25–29], are reported to prevent VT/VF in both syndromes [26,30,31]. Despite its ameliorative effects, quinidine, by virtue of its action to block I_{Kr}, prolongs the QT interval and predisposes to development of life-threatening Torsade de Pointes

(TdP) arrhythmias. High plasma levels of quinidine are required to avoid this side effect, but these high doses cause approximately 50% of patients to develop serious gastrointestinal side effects. AR-787 does not exhibit these undesirable effects. Cilostazol is not always effective, and the other pharmacological approaches mentioned have limitations as well. Thus, there is a critical need for additional safe and effective agents. Our findings point to AR-787 as a promising candidate in the armamentarium available for the treatment of JWS.

Study limitations

While the pharmacological models that we employ may not precisely mimic the effect of the diverse genetic defects underlying the clinical syndromes, "more realistic" transgenic large mammal models do not exist. Our observations using the wedge models over 20 years ago suggested for the first time that an early repolarization pattern in the ECG, previously thought to be totally benign, can reveal the presence of a substrate for the development of malignant arrhythmias [1]. These experimental approaches have been instrumental in identifying many novel therapies for both BrS and ERS [3], including use of quinidine and catecholamines, which are widely used in the clinic today in the management of these life-threatening syndromes [2,3]. These experimental models have also recently been validated using a whole-heart model of the J wave syndromes [6].

Author Contributions

Conceptualization: Mena Abdelsayed, Charles Antzelevitch.

Data curation: Hector Barajas-Martinez, Robert Cox, Victoria M. Robinson, Bence Patocskai, Mena Abdelsayed, Charles Antzelevitch.

Formal analysis: Robert Cox, Joseph Jung, Mohamed Fouda, Mena Abdelsayed, Peter C. Ruben, Charles Antzelevitch.

Funding acquisition: Charles Antzelevitch.

Investigation: José M. Di Diego, Hector Barajas-Martinez, Robert Cox, Victoria M. Robinson, Joseph Jung, Mohamed Fouda, Bence Patocskai, Mena Abdelsayed, Peter C. Ruben, Charles Antzelevitch.

Methodology: José M. Di Diego, Hector Barajas-Martinez, Robert Cox, Victoria M. Robinson, Bence Patocskai, Mena Abdelsayed, Charles Antzelevitch.

Project administration: Peter C. Ruben, Charles Antzelevitch.

Resources: Charles Antzelevitch.

Supervision: Charles Antzelevitch.

Validation: José M. Di Diego, Hector Barajas-Martinez, Victoria M. Robinson, Bence Patocskai, Mena Abdelsayed, Peter C. Ruben, Charles Antzelevitch.

Writing – original draft: Hector Barajas-Martinez, Bence Patocskai, Mena Abdelsayed, Charles Antzelevitch.

Writing – review & editing: José M. Di Diego, Hector Barajas-Martinez, Robert Cox, Victoria M. Robinson, Mena Abdelsayed, Peter C. Ruben, Charles Antzelevitch.

References

1. Antzelevitch C, Yan GX. J wave syndromes. Heart Rhythm. 2010; 7(4):549–58. https://doi.org/10.1016/j.hrthm.2009.12.006 PMID: 20153265

2. Antzelevitch C, Yan GX, Ackerman MJ, Borggrefe M, Corrado D, Guo J, et al. J-Wave syndromes expert consensus conference report: Emerging concepts and gaps in knowledge. Heart Rhythm. 2016. Epub 2016/07/18. https://doi.org/10.1016/j.hrthm.2016.05.024 PMID: 27423412.
3. Yan GX, Antzelevitch C. Cellular basis for the Brugada syndrome and other mechanisms of arrhythmogenesis associated with ST-segment elevation. Circulation. 1999; 100(15):1660–6. https://doi.org/10.1161/01.cir.100.15.1660 PMID: 10517739
4. Koncz I, Gurabi Z, Patocskai B, Panama BK, Szel T, Hu D, et al. Mechanisms underlying the development of the electrocardiographic and arrhythmic manifestations of early repolarization syndrome. J Mol Cell Cardiol. 2014; 68C:20–8. https://doi.org/10.1016/j.yjmcc.2013.12.012 PMID: 24378566
5. Di Diego JM, Sicouri S, Myles RC, Burton FL, Smith GL, Antzelevitch C. Optical and electrical recordings from isolated coronary-perfused ventricular wedge preparations. J Mol Cell Cardiol. 2013; 54(1):53–64. https://doi.org/10.1016/j.yjmcc.2012.10.017 PMID: 23142540
6. Di Diego JM, Patocskai B, Barajas-Martinez H, Borbáth V, Ackerman MJ, Burashnikov A, et al. Acacetin suppresses the electrocardiographic and arrhythmic manifestations of the J wave syndromes. PloS one. 2020; 15(11):e0242747. Epub 2020/11/25. https://doi.org/10.1371/journal.pone.0242747 PMID: 33232375; PubMed Central PMCID: PMC7685455.
7. Barajas-Martinez H, Hu D, Ferrer T, Onetti CG, Wu Y, Burashnikov E, et al. Molecular genetic and functional association of Brugada and early repolarization syndromes with S422L missense mutation in KCNJ8. Heart Rhythm. 9(4):548–55. https://doi.org/10.1016/j.hrthm.2011.10.035 PMID: 22056721.
8. Barajas-Martinez H HV, Chamberland C, Blais Roy M-J, Fecteau MH, Cordeiro JM, Dumaine R. Larger dispersion of INa in female dog ventricle as a mechanism for gender-specific incidence of cardiac arrhythmias. Cardiovascular research. 2009; 81(1):82–9. https://doi.org/10.1093/cvr/cvn255 PMID: 18805783
9. Gussak I, Antzelevitch C, Bjerregaard P, Towbin JA, Chaitman BR. The Brugada syndrome: clinical, electrophysiologic and genetic aspects. J Am Coll Cardiol. 1999; 33(1):5–15. https://doi.org/10.1016/s0735-1097(98)00528-2 PMID: 9935001
10. Antzelevitch C, Yan GX. Cellular and ionic mechanisms responsible for the Brugada syndrome. JElectrocardiol. 2000; 33 Suppl:33–9. https://doi.org/10.1054/jelc.2000.20321 PMID: 11265734
11. Shimizu W, Aiba T, Antzelevitch C. Specific therapy based on the genotype and cellular mechanism in inherited cardiac arrhythmias. Long QT syndrome and Brugada syndrome. CurrPharmDes. 2005; 11(12):1561–72. https://doi.org/10.2174/1381612053764823 PMID: 15892662
12. Antzelevitch C, Fish JM. Therapy for the Brugada syndrome. Handb Exp Pharmacol. 2006;(171):305–30. https://doi.org/10.1007/3-540-29715-4_12 PMID: 16610350
13. Szel T, Koncz I, Antzelevitch C. Cellular mechanisms underlying the effects of milrinone and cilostazol to suppress arrhythmogenesis associated with Brugada syndrome. Heart rhythm: the official journal of the Heart Rhythm Society. 2013; 10(11):1720–7. https://doi.org/10.1016/j.hrthm.2013.07.047 PMID: 23911896
14. Patocskai B, Antzelevitch C. Novel Therapeutic Strategies for the Management of Ventricular Arrhythmias Associated with the Brugada Syndrome. Expert Opin Orphan Drugs. 2015; 3(6):633–51. Epub 2015/01/01. https://doi.org/10.1517/21678707.2015.1037280 PMID: 27559494; PubMed Central PMCID: PMC4993532.
15. Antzelevitch C, Yan GX. J-wave syndromes: Brugada and early repolarization syndromes. Heart Rhythm. 2015; 12(8):1852–66. Epub 2015/04/15. https://doi.org/10.1016/j.hrthm.2015.04.014 PMID: 25869754.
16. Argenziano M, Antzelevitch C. Recent advances in the treatment of Brugada syndrome. Expert Rev Cardiovasc Ther. 2018; 16(6):387–404. Epub 2018/05/15. https://doi.org/10.1080/14779072.2018.1475230 PMID: 29757020; PubMed Central PMCID: PMC6330094.
17. Yoon JY, Ahn SH, Oh H, Kim YS, Ryu SY, Ho WK, et al. A novel Na+ channel agonist, dimethyl lithospermate B, slows Na+ current inactivation and increases action potential duration in isolated rat ventricular myocytes. BrJ Pharmacol. 2004; 143(6):765–73. https://doi.org/10.1038/sj.bjp.0705969 PMID: 15504759
18. Fish JM, Welchons DR, Kim YS, Lee SH, Ho WK, Antzelevitch C. Dimethyl lithospermate B, an extract of danshen, suppresses arrhythmogenesis associated with the Brugada syndrome. Circulation. 2006; 113(11):1393–400. https://doi.org/10.1161/CIRCULATIONAHA.105.601690 PMID: 16534004
19. Shimizu W, Kamakura S. Catecholamines in children with congenital long QT syndrome and Brugada syndrome. J Electrocardiol. 2001; 34 Suppl:173–5. https://doi.org/10.1054/jelc.2001.28864 PMID: 11781952
20. Suzuki H, Torigoe K, Numata O, Yazaki S. Infant case with a malignant form of Brugada syndrome. Journal of cardiovascular electrophysiology. 2000; 11:1277–80. https://doi.org/10.1046/j.1540-8167.2000.01277.x PMID: 11083249

21. Ohgo T, Okamura H, Noda T, Satomi K, Suyama K, Kurita T, et al. Acute and chronic management in patients with Brugada syndrome associated with electrical storm of ventricular fibrillation. Heart Rhythm. 2007; 4(6):695–700. https://doi.org/10.1016/j.hrthm.2007.02.014 PMID: 17556186

22. Watanabe A, Fukushima KK, Morita H, Miura D, Sumida W, Hiramatsu S, et al. Low-dose isoproterenol for repetitive ventricular arrhythmia in patients with Brugada syndrome. EurHeart J. 2006; 27(13):1579–83. https://doi.org/10.1093/eurheartj/ehl060 PMID: 16760208

23. Hermida JS, Denjoy I, Clerc J, Extramiana F, Jarry G, Milliez P, et al. Hydroquinidine therapy in Brugada syndrome. J Am Coll Cardiol. 2004; 43(10):1853–60. https://doi.org/10.1016/j.jacc.2003.12.046 PMID: 15145111

24. Belhassen B, Viskin S. Pharmacologic approach to therapy of Brugada syndrome: quinidine as an alternative to ICD therapy? In: Antzelevitch C, Brugada P, Brugada J, Brugada R, editors. The Brugada Syndrome: From Bench to Bedside. Oxford: Blackwell Futura; 2004. p. 202–11.

25. Tsuchiya T, Ashikaga K, Honda T, Arita M. Prevention of ventricular fibrillation by cilostazol, an oral phosphodiesterase inhibitor, in a patient with Brugada syndrome. JCardiovascElectrophysiol. 2002; 13 (7):698–701.

26. Iguchi K, Noda T, Kamakura S, Shimizu W. Beneficial effects of cilostazol in a patient with recurrent ventricular fibrillation associated with early repolarization syndrome. Heart Rhythm. 2013; 10(4):604–6. https://doi.org/10.1016/j.hrthm.2012.11.001 PMID: 23142636

27. Agac MT, Erkan H, Korkmaz L. Conversion of Brugada type I to type III and successful control of recurrent ventricular arrhythmia with cilostazol. ArchCardiovasc Dis. 2013. https://doi.org/10.1016/j.acvd.2012.06.008 PMID: 23791603

28. Hasegawa K, Ashihara T, Kimura H, Jo H, Itoh H, Yamamoto T, et al. Long-term pharmacological therapy of Brugada syndrome: is J-wave attenuation a marker of drug efficacy? InternMed. 2014; 53 (14):1523–6. https://doi.org/10.2169/internalmedicine.53.1829 PMID: 25030565

29. Shinohara T, Ebata Y, Ayabe R, Fukui A, Okada N, Yufu K, et al. Combination therapy of cilostazol and bepridil suppresses recurrent ventricular fibrillation related to J-wave syndromes. Heart Rhythm. 2014; 11(8):1441–5. https://doi.org/10.1016/j.hrthm.2014.05.001 PMID: 24813378

30. Nam GB, Kim YH, Antzelevitch C. Augmentation of J waves and electrical storms in patients with early repolarization. NEnglJ Med. 2008; 358(19):2078–9. https://doi.org/10.1056/NEJMc0708182 PMID: 18463391

31. Haissaguerre M, Chatel S, Sacher F, Weerasooriya R, Probst V, Loussouarn G, et al. Ventricular fibrillation with prominent early repolarization associated with a rare variant of KCNJ8/KATP channel. Journal of cardiovascular electrophysiology. 2009; 20(1):93–8. https://doi.org/10.1111/j.1540-8167.2008.01326.x PMID: 19120683.
